# Financial burden and social implications of chronic liver disease in a patient population group in Pakistan

**DOI:** 10.12669/pjms.40.7.7976

**Published:** 2024-08

**Authors:** Adnan Salim, Muhammad Omer Farooq, Sonia Saleem, Kashif Malik

**Affiliations:** 1 Adnan Salim, FCPS FRCP (Glasgow) Associate Professor, Gastroenterology & Hepatology, Shaikh Zayed Hospital & Postgraduate Medical Institute, Lahore, Pakistan; 2 Muhammad Omer Farooq, FCPS Senior Registrar, Gastroenterology & Hepatology, Shaikh Zayed Hospital & Postgraduate, Medical Institute, Rahim Yar Khan, Pakistan; 3 Sonia Saleem, FCPS Gastroenterology Consultant, Gastroenterologist & Hepatologist, DHA Medical Center, Lahore, Pakistan; 4 Kashif Malik, FCPS Professor, Gastroenterology & Hepatology, Shaikh Zayed Hospital & Postgraduate Medical Institute, Lahore, Pakistan

**Keywords:** Chronic Liver Disease, Financial burden, Social implications

## Abstract

**Objective:**

To assess economic and social issues faced by cirrhotic patients & its financial burden for developing nations like Pakistan.

**Method:**

This cross-sectional study was carried out at the Department of Gastroenterology & Hepatology, Shaikh Zayed Hospital, Lahore, Pakistan during the period between July & December 2019. Patients with liver cirrhosis were recruited and information regarding disease, financial status, treatment expenses & dependency was recorded.

**Results:**

A total of 450 patients were recruited, 272 (60%) were males & 178 (40%) were females, with mean age 55.4±6.2 years. HCV was cause of cirrhosis in 86% of cases, 65% were diagnosed incidentally and 39.6% were illiterate. About 82.7% were urban while only 28.7% own their own home. Co-morbid conditions including diabetes, hypertension & ischemic heart disease were present in 54% of cases. Monthly income was <PKR 45,000 in 23% of cases while 47% were non-earning.

**Conclusions:**

Our study shows the financial difficulties & dependency faced by patients with liver cirrhosis. Aggressive national screening is required to discover infected patients before cirrhosis develops.

## INTRODUCTION

Pakistan has one of the highest viral hepatitis prevalence worldwide.^1^,^2^ A large percentage of patients develop chronic liver disease (CLD).^3^,^4^ This is because hepatitis remains asymptomatic and goes undetected in a vast majority due to lack of national screening policy. Timely anti-viral therapy is not initiated and cirrhosis has developed by the time a patient develops symptoms. Besides viruses, other, rarer, causes of cirrhosis in Pakistan are alcohol (3.2%), primary biliary cholangitis (2.1%), Wilson’s disease and haemochromatosis (1.05%).^5^ Pakistan is a developing country with average Gross Domestic Product(GDP) of 284 billion US, per capita income 1,505 USD and over 24% populace below poverty line.^6^ It is not a welfare state, and treatment, though subsidized, is not completely. Some degree of free services are provided in some government hospitals but this does not extend to higher end medications, procedures and investigations.

Complications of cirrhosis lead to repeated admissions requiring expensive medications and high-end procedures. Liver transplantation (Pak rupees/PKR 4-5 million) is the only cure and remains unaffordable for the vast majority. Hence, nearly all liver transplants carried out at government transplant centres are financed by the government. The financial implications are tremendous.^1^ If an earning family member is ill, their earning capacity is compromised. If a non-earning member is afflicted, significant earnings are diverted to treatment. Difficult decisions including selling property and taking loans are often taken.

Dependency of patients is another dilemma. The performance of patients within households is compromised, putting additional burden on family. CLD thus carries extreme financial and social implications, regardless of developing/developed countries and healthcare structures.^7^,^8^ It is therefore imperative, for Pakistan, that the socioeconomic impact of CLD is investigated. This will assist in decisions prioritizing national health policies and services. The objective of this study was to investigate the social and financial challenges of liver cirrhosis faced by patients and their families. The working hypothesis was that chronic liver disease constitutes a major social and financial burden for patients.

### Operational Definitions:

- ***Chronic Liver Disease:*** Chronic liver disease refers to disease of the liver which lasts over a period of six months. It involves a process of progressive destruction and regeneration of the liver parenchyma leading to fibrosis and cirrhosis.^2^
- Complications of liver cirrhosis including:
- ***Ascites:*** Ascites is a gastroenterological term for accumulation of fluid in the peritoneal cavity that exceeds 25ml.^2^
- ***Portal hypertension:*** Portal hypertension is hypertension (high blood pressure) in the hepatic portal system, which are the portal vein and its branches, which drain from most of the intestines to the liver. Portal hypertension is defined as a hepatic venous pressure gradient equal to or greater than six mmHg.^9^
- ***Portosystemic encephalopathy:*** Portosystemic encephalopathy is a neuropsychiatric syndrome.^9^
- Hepatocellular carcinoma (HCC) was diagnosed on basis of ultra-sound findings of mass with vascularity inside it and on triphasic contrast enhanced computerised tomography (CECT) scan of the abdomen.^10^

## METHODS

The study was a cross-sectional survey, done at Shaikh Zayed Federal Postgraduate Medical Institute, Lahore, Pakistan, between July and December 2019. A sample size of 375 was calculated by 95% confidence level and 5% margin of error with expected percentage of financial burden, i.e., 42%, in patients with CLD.^6^ We report data from 450 patients.

All adult patients suffering from complications of CLD: spontaneous bacterial peritonitis (SBP), ascites, portal hypertension, variceal bleeding, portosystemic encephalopathy-(PSE) and hepatocellular carcinoma (HCC), admitted to our unit were recruited. Patients with advanced chronic diseases of other organ systems needing repeated admissions such as renal failure requiring haemodialysis, congestive cardiac failure, severe chronic obstructive pulmonary disease and active malignancies other than HCC were excluded from the study. Patients with stable, non-complicated co-morbidities, such as diabetes, hypertension, and ischemic heart disease, controlled with medications, were included in the study.

The patients were questioned regarding residence, education, cause and duration of cirrhosis, how cirrhosis was initially discovered, number of admissions with expenditure per admission, outpatient follow-up, knowledge regarding disease, presence of HCC and co-morbidities, employment, financial constraint on self or family and dependency at home. All findings were recorded through a specially designed proforma. Data was analysed using SPSS 22.

### Ethical Approval

After obtaining ethical approval from institutional review board (Approval Reference: SZMC/IRB/Internal/338/2021, dated 28^th^ June 2021) informed consent was also obtained from those included..

## RESULTS

Out of 450 patients recruited, 272 (60.4%) were male and 178 (39.6%) females. Average age was 55.4±6.2 years. 373 (82.9%) were married while 70 (15.6%) women were widowed. 7(1.6%) patients were single. Details regarding demography, education, residence & co-morbidities of selected patients in given in Table-I.

**Table-I:** Details of demography, education, residence & co-morbidities of selected cohort.

***Demography***	
Total recruited	450
Males	272 (60.4%)
Females	178 (39.6%)
Mean age ±SD years	55.4 ±6.2
***Education Status***	
Illiterate	178 (39.6%)
Primary education	75 (16.7%)
Middle level	64 (14.2%)
Secondary level	59 (13.1%)
Higher Secondary	37 (8.2%)
Bachelor	31 (6.9%)
Masters	6 (1.3%)
***Residence***	
Urban	372 (82.7%)
Rural	78 (17.3%)
Own Home	129 (28.7%)
Rented home	44 (9.8%)
Shared home	277 (61.6%)
***Family members residing in same home***	
≤5 persons	74 (16.4%)
6-10 persons	280 (62.2%)
11-15 persons	65 (14.4%)
≥16 persons	31 (6.9%)
***Co-Morbidities n=242 (54%)***	
Diabetes Mellitus	101 (22.4%)
Hypertension	35 (5.8%)
Diabetes plus Hypertension	102(22.7%)
Ischemic heart disease	2 (0.4%)

Majority of patients, 324 (72.0%) sought healthcare services in their native towns before coming to our centre, and all of them reported better care as reason for seeking treatment at our centre. Cirrhosis was diagnosed in last one year in 119 (26.5%) patients, while in 237 (52.7%) it was diagnosed in last three years. Non-specific symptoms of weakness and fatigue were present in 293 (65%) before diagnosis of cirrhosis. While 131 (29.2%) were diagnosed after suffering complication of liver cirrhosis, e.g., variceal bleed, PSE, SBP & HCC. HCV was present in 388 (86.2%) patients. HBV in 20 (4.4%). HCV & HBV in 21 (4.7%), HCV & alcohol in 9 (2.0%). Alcohol in 8 (1.8%) and unknown causes in 4 (0.9%). Twenty-six (5.8%) were diagnosed on routine screening for viral hepatitis (prior to blood donation or local screening camp). Detailed results are presented in tabulated form in Table-II.

**Table-II:** Detailed results in tabulated form.

***Status***	
HCC	170 (37.8%)
HBV Vaccinated	10 (2.2%)
Herbal Medication Use	167 (37.1%)
Treatment Naïve	197 (43.8%)
***Follow-up Schedule***	
Monthly	174 (38.7%)
Bi-monthly	98 (21.8%)
Three Months	19 (4.2%)
Yearly	2 (0.4%)
Irregular	162 (36.0%)
***Knowledge regarding disease***	
Good	43 (9.6%)
Mediocre	270 (60.0%)
Poor	137 (30.4%)
***Treatment Compliance***	
Adequate	17 (3.8%)
Inadequate	433 (96.2%)
Dependency	
Limited ability to work	448 (99.6%)
Dependent on children	276 (61.3%)
Dependent on spouses	81 (18.0%)
Dependent on relatives	83 (18.4%)
Dependent on friends/neighbours	6 (1.3%)
***Profession***	
Labourers	107 (24.0%)
Self Employed	83 (18.5%)
Farmers	33 (7.3%)
Government employee	30 (6.7%)
Private employee	18 (4.0%)
***Admission History***	
> Ten times	35 (7.7%)
Up to Five times	258 (57.3%)
Once before	157 (34.9%)
During previous three months	420 (93.4%)
***Monthly Income***	
PKR ≤45,000	105 (23.0%)
PKR >45,000	38 (8.4%)
Non-earning family member	214 (47.6%)
Sole earning family member	118 (26.0%)

The expenses occurred during admission were reported as PKR 50,000.00 to 150,000.00 per admission. Seventy (15.5%) reported stable, regular earnings from agricultural land, rented properties or investments. 359 (80%) patients reported requiring regular financial help from relatives. To meet the expenses of admission/treatment, 113 (25.0%) patients liquidated personal property/belongings, 49 (10.9%) sold personal jewellery, 29 (6.4%) sold other built-up properties, 27 (6.0%) sold non-built-up properties including agricultural land and 8 (1.8%) sold their homes.

Treatment support in form of health insurance was available to none, two (0.4%) patients had employer-supported treatment, six (1.3%) patients had full reimbursement of treatment expenses & 7 (1.6%) had employer reimbursement for only inpatient expenses.

Out of 220 (48.9%) who were liver transplant candidates only 10 (2.0%) could afford transplantation, while 230 (51.1%) were unfit due to advanced HCC, age and complicated co-morbid conditions.

## DISCUSSION

Our study highlights important basic problems related to liver disease in our country: inadequate screening and late discovery of cirrhosis with a downhill socioeconomic path. Hepatitis takes decades to progress to cirrhosis but over half our patients discovered advanced liver disease only in past three years. It is therefore important to elaborate each individual finding in our results with respect to regional and global observations to understand its significance.

The majority were diagnosed at average age of 55 years. This age group is extremely productive, socially and professionally. It is important to consider the family structure in Pakistani society whereby nearly all female patients in our study were housewives. While disease in such patients may not adversely affect the professional population, the roles these women play in society both in looking after homes and upbringing children is invaluable, considering male members are out for work routinely up to 10-14 hours a day.

Literacy amongst our patients was alarmingly low. Nearly 40% were completely illiterate. An unacceptably small number had higher secondary school education while percentage with bachelor/master education was even lower. These are far lower than neighbouring countries like India (76%) Sri Lanka (92%) and China (99%). These figures speak of greater national problems. Inadequate literacy automatically means low health awareness, poor understanding of disease and its associated responsibilities.^11^

Over 80% patients were urban dwellers, nearly a third lived in ancestral homes. Hence, we observed large numbers of people sharing homes: over half reported six to ten family members under one roof. This is a combination of inflation and increased populations with preference for urban settlement as also seen in regional countries like India and Bangladesh.^12^

Although over 70% patients had consulted local physicians, all reported inadequate/unsatisfactory health expertise as primary reason for coming to our facility. This “urban health seeking behaviour” is also evident in other developing countries, figures up to 70% seen in an Iranian study and similar figures seen in various studies carried out in India.^13^ The reasons quoted for avoiding local primary health services both in our study population and those in the above mentioned studies have been lack of satisfaction and trust with local healthcare providers. Many primary/secondary health facilities are also failing in providing basic management, which is very much possible with targeted training of staff, for such patients. This problem is universal and patients suffering from diseases of all organ systems are faced with inadequately equipped/trained services.

Another issue remains of patient satisfaction. There are also cases whereby local facilities have provided requisite care, however mental satisfaction of going to large hospitals in a major city also increases tertiary workload. For example, managing SBP, PSE or HRS, especially in patients with advanced HCC, destined for palliative care, is well within capacity of many secondary care institutes. However, lack of counselling on part of tertiary teams in instilling confidence for seeking local care as well as reluctance of patients means even simple procedures like therapeutic paracentesis land in tertiary centres. We have experienced the latter first hand as our regular follow-up patients prefer to travel to our facility rather than seek basic treatment nearer to home.

Nearly half our patients had co-morbidities including diabetes, hypertension, ischemic heart disease and chronic obstructive pulmonary disease (COPD), further burdening treatment. Furthermore, over a third had already developed hepatocellular carcinoma, the most lethal complication of cirrhosis. HCC carries enormous emotional/financial consequences with treatments like microwave ablation (PKR 260,000.00 per session), TACE (PKR 300,000.00 to 600,000.00 per session) and surgical resection being unaffordable for a vast majority.^14^

Another startling discovery was extremely low rate of vaccination against HBV. Other studies have highlighted this problem in general population and healthcare workers. For a country among top affected by HCV worldwide, the last thing needed is a spot on the HBV podium. This clearly highlights a major shortcoming (or lack) of adult screening/vaccination and needs aggressive countering. Our neighbour India, despite not having acceptable adult vaccination rates, still fares much better with over 20% adult HBV vaccination rates.^15^,^16^

Over a quarter of patients had used alternative/herbal medicine, formulated by quacks. The detrimental effects on liver are known but not investigated in this study.^17^ Nearly half the patients had not received antiviral therapy. This again is failure at level of primary care and screening. Unfortunate, as treatment of viral hepatitis has become extremely affordable with generic drugs yielding excellent results. These medicines are available free of cost at government hospitals. In open market, prices are well within reach of common people.^18^

Nearly a third of patients had inadequate follow up in the outpatient clinics. Reasons cited were financial, intentional and inadequate counselling on importance of follow-up. Nearly all patients had mediocre to poor knowledge about their disease, reflecting failure in health education at community level and counselling at healthcare facility level. Regardless of poor knowledge and inadequate follow-up, treatment compliance was surprisingly satisfactory in our patients, as also observed in another local study.^19^

Disease related frailty/dependency was devastating. Reliance on close family was nearly universal for basic activities. Nearly three quarters reported total loss of ability to perform any productive domestic/professional tasks. Patients could not cook, clean, independently use toilet or bathe. A close-knit family system highlighted important roles played by children, grandchildren and spouses in patient care. This also reflects total absence of any community services aiding patients. The burden on all family members is tremendous with even one chronically ill member. Hence social services are a boon to both those living alone or with family. Such services also do not necessarily need salaried members. Instead, volunteers aiding deserving people in their own neighbourhoods, coordinating with local governments, are an effective solution. Unfortunately, dependency due to cirrhosis, both at home and in hospital, with relatives assisting during the duration of admission has never been investigated in Pakistan. Attendants living with admitted patients is common in our hospitals as they have to perform tasks from bringing medicines to basic assistance of patient. These attendants are healthy individuals who do so at expense of domestic, professional and academic responsibilities. This burden is global and affects developed, developing and underdeveloped nations alike.^1^,^19^

The economic impact of liver cirrhosis was also obvious. The largest number of patients, a quarter, were labourers, the most financially vulnerable social segment. All patients had been repeatedly admitted to hospital with complications of cirrhosis. Despite treatment being government subsidized at our hospital, each patient spent about PKR 85,000.00 to 250,000.00 per admission. The monthly earning capacity puts this figure into perspective: a quarter of patients earned more than PKR 45,000 per month. And around half the patients earned below PKR 45000 per month. A huge number, 80%, of patients (including all women, bar one, being housewives), were financially dependent on family members for all needs. Further humbling facts: a low number (15%) of patients who had a steady income from sources that remained unaffected by their own inability to work; only 3% having some sort of insurance or employer support/reimbursement for treatment (with half having only inpatient reimbursement and none for outpatient expense); a quarter of patients having to sell belongings/property to pay treatment cost for their chronic disease; another quarter benefitting from financial aid from relatives/friends. Other Asian countries such as Thailand report similar observations.^20^,^21^,^22^

Despite nearly half our patients fulfilling criteria for liver transplantation, only 2% actually afforded it. The other half had advanced disease and factors that made them unfit for transplantation, again highlighting late discovery of advanced complicated disease unfit for any curative treatment, thereby condemning to a long, expensive and painful course with repeated admissions.^23^

### Limitations

Although our study was limited by a small sample size from only one institute, it concentrated only on patients presenting to our tertiary care facility with resultant bias in awareness and some limited appreciation of treatment costs. A larger study encompassing more tertiary care liver units, especially from other cities in the country will show, in our observation, even more humbling figures.

## CONCLUSION

Our study highlights major shortcomings in preventive health policies and practical non-existence of “community medicine”: Evident by inadequate community education regarding disease and reporting disease statistics to higher health authorities regarding a disease which is epidemic for decades.

The failure is in detecting disease early, in timely referral for treatment and in administering vaccination to adults. Although HBV vaccination is part of the national vaccination program for children, no such policy exists for adults. Aggressive national door to door screening to identify those needing treatment/vaccinations is mandatory. We must detect and treat before cirrhosis develops. Staggering financial difficulties and dependency are faced by patients and their families. These strike at the very core of social fabric and lead to enormous emotional and financial strain.

### Authors’ Contributions:

**AS:** Conceived the study, writeup of manuscript and gave final approval of manuscript for publication. **MOF:** Did data collection and initial writeup of manuscript, also responsible for data integrity. **SS:** Did data collection and statistical analysis, also responsible for data integrity.

**KM:** Final approval of manuscript for publication.
